# Synthesis and Thermochromic Properties of Cr-Doped Al_2_O_3_ for a Reversible Thermochromic Sensor

**DOI:** 10.3390/ma10050476

**Published:** 2017-04-28

**Authors:** Duy Khiem Nguyen, Heesoo Lee, In-Tae Kim

**Affiliations:** 1 Department of Civil Engineering, Pusan National University (PNU), 30 Jangjeon-dong, Geumjeong-gu, Busan 46241, Korea; khiemduynguyen2000@yahoo.com; 2 Department of Materials Science and Engineering, Pusan National University (PNU), 30 Jangjeon-dong, Geumjeong-gu, Busan 46241, Korea; heesoo@pusan.co.kr

**Keywords:** reversible thermochromic sensors, Cr-doped alumina, thermochromic materials, thermochromism

## Abstract

An inorganic thermochromic material based on Cr-doped Al_2_O_3_ is synthesized using a solid-state method. The crystal structure, chemical composition, and morphology of the synthesized material are analyzed using X-ray diffraction, scanning electron microscopy coupled with an energy-dispersive X-ray spectrometer, and Fourier transform infrared (FT-IR) spectroscopy. The color performances of the synthesized material are analyzed using a UV-VIS spectrometer. Finally, the thermochromism exhibited by the powdered samples at high temperatures is investigated. The material exhibits exceptional thermochromic property, transitioning from pink to gray or green in a temperature range of 25–600 °C. The change in color is reversible and is dependent on the surrounding temperature and chromium concentration; however, it is independent of the exposure time. This novel property of Cr-doped Al_2_O_3_ can be potentially employed in reversible thermochromic sensors that could be used not only for warning users of damage due to overheating when the environmental temperature exceeds certain limits, but also for detecting and monitoring the temperature of various devices, such as aeronautical engine components, hotplates, and furnaces.

## 1. Introduction

The ability to change color at a predetermined temperature threshold is very useful for a temperature-sensing thermochromic material. The color change property of thermochromic materials can be employed to alert about visible damage due to overheating; further, the change in color could indicate irregularity, and it can be used to monitor engine component temperatures or to warn users if the environmental temperature exceeds certain limits [1]. Hence, such materials have received much attention because of their potential applications as temperature sensors in a wide range of devices, such as aeronautical engine components [2,3], household appliances [4], hotplates, and furnaces [5]. 

Thermochromic sensors can be divided into two categories: irreversible thermochromic materials and reversible thermochromic materials. In the first classification, the materials exhibit an irreversible color change based on the peak temperature of the surrounding environment. The change in color cannot be reversed on cooling, thus providing permanent records, which can be visualized offline [2]. In the second classification, the materials exhibit a reversible color change with respect to a change in the temperature. The change in color is reversible in the heating–cooling cycles, wherein the material regains its original color after cooling.

Various thermochromic materials have been used as thermochromic sensors, such as iron yellow, basic cupric carbonate, zinc white [1], Mn^2+^-doped Zn_3_(PO_4_)_2_ [6], and Cr-doped Al_2_O_3_ [7]. Recently, Wang et al. reviewed the recent progress in fabrication of vanadium oxide (VO_2_) which could be applied in thermochromic smart windows. Thermochromic performance of VO_2_ is largely dependent on the synthesis method and growth control. VO_2_ thin films with nanostructures are desired because they can improve the thermochromic properties. However, the transition temperature (T_c_) of VO_2_ is too low (T_c_ of bulk VO_2_ is only 68 °C) which cannot be used as a temperature sensor for monitoring high temperatures (≥200 °C), such as of aeronautical engine components, hotplates, or furnaces [8]. More recently, Ke et al. prepared SiO_2_/VO_2_ 2D photonic crystals for thermochromic smart window applications. By selectively blocking visible light, both transmittance and reflectance could be tuned, the photonic crystals showed distinct color change from red, to green, to blue. Simultaneously, these photonic crystals maintained IR transmission at low temperature, but strongly attenuated IR transmission at high temperature, which is necessary for thermochromic smart glasses. However, similar to the VO_2_ system, this SiO_2_/VO_2_ 2D photonic crystal system can be used only at low temperatures (≤100 °C) [9]. Željka Rašković-Lovre et al. have studied the thermochromic and photochromic color change in Mg-Ni-H thin films deposited by reactive magnetron sputtering [10]. A change in optical properties was observed at T = 200 °C. Moreover, a color change from yellow to red was also observed as the temperature increased from 20 to 200 °C.

Among the above-mentioned thermochromic materials, Cr-doped Al_2_O_3_ is an attractive candidate, which can be used as a reversible thermochromic sensor. When the Cr^3+^ ions replace the Al^3+^ ions in the Al_2_O_3_, the resulting Cr-doped Al_2_O_3_ material is referred to as ruby or ruby solid solution [11,12]. If the chromium concentration in the compounds is low, the color of the ruby is pink. At high chromium concentration, the color of the compounds is green [4]. The advantages of ruby are high heat resistance, high melting point, high mechanical strength, high transparency, and excellent chemical stability [13]. More importantly, the compounds can exhibit a remarkable reversible thermochromic phenomenon [12]. On heating, the color of ruby changes from pink at low temperatures to green at high temperatures. This change is observed in a wide range of temperatures ranging from 200 to 900 °C [4]. The color change of the Cr-doped Al_2_O_3_ can be explained based on the ligand field theory of transition metal complexes. The colors of these transition metal complexes are because of the d–d bands, or because the electronic transitions between the d-orbitals split under the electric field of the ligands. This splitting of the d-orbitals is the origin of the red color of the Cr-doped Al_2_O_3_. At low temperatures, a Cr^3+^ ion is being squeezed into an octahedral cage of O^2−^ ions, which is too small. At high temperatures, the chemical bonds in this compound expand, and the Cr^3+^ ions become more relaxed, regaining their proper green color, at the expense of the Al^3+^ ions in the ligand cages which are now too large. The competition between these two cations to be in a proper-sized cage is, thus, the origin of this thermochromism [14]. The threshold of the color-transition temperature can be varied depending on the concentration of chromium doped in the Al_2_O_3_ lattice: 5% Cr_2_O_3_–95% Al_2_O_3_ turns from red to green at approximately 250 °C, and 10% Cr_2_O_3_–90% Al_2_O_3_ turns from red to green/gray in the range of 400–450 °C [7].

Many methods are used to prepare Cr-doped Al_2_O_3_, e.g., chemical vapor deposition [15,16], the combustion method [17,18,19,20,21], conventional solid–state method [22,23,24,25], sol–gel method [26,27,28], hydrothermal method [29,30], pulsed electric-current sintering process [31,32], and microwave-solvothermal method [33,34]. Recently, the solid-state method has been widely used to synthesize colored Cr-doped ceramic pigments [23,24,25]. This method involves the mixture dispersion of the metal oxides in a planetary ball mill and, subsequently, heat treatment of the mixtures at temperatures above 1300 °C for the solid-state reaction [23].

Although many studies have been conducted to analyze the properties of Cr-doped Al_2_O_3_ systems and their applications in ceramic pigments [35,36], only a few studies on the synthesis of Cr-doped Al_2_O_3_ for application in thermochromic sensors have been reported [4,7]. In this study, Cr-doped Al_2_O_3_ with chromium-oxide compositions of 5, 10, 20, and 40 wt % were prepared using the solid-state method under air, up to 1600 °C, to analyze their structural evolution and properties. The thermochromism of the compounds at 200, 400, and 600 °C, which may be applied for reversible thermochromic sensors, is also investigated. These sensors can be used not only for visible damage warning of overheating, but also for monitoring the temperature of various devices.

## 2. Results and Discussion

### 2.1. Synthesis

**Figure 1:**
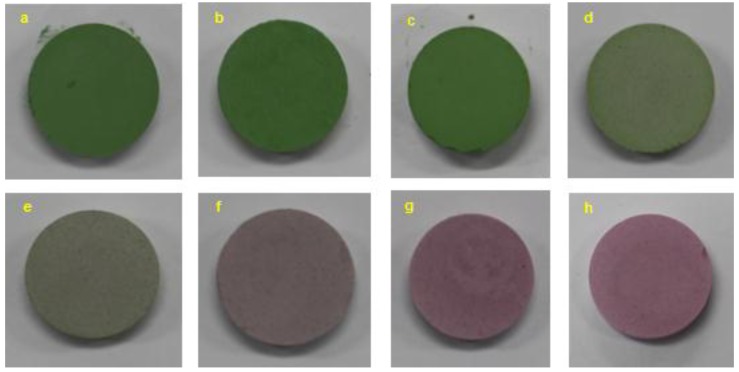
Images of 10 wt % Cr-doped Al_2_O_3_ samples annealed at various temperatures for 6 h: (**a**) as prepared; (**b**) 1000 °C; (**c**) 1100 °C; (**d**) 1200 °C; (**e**) 1300 °C; (**f**) 1400 °C; (**g**) 1500 °C; and (**h**) 1600 °C.

Figure 1 shows the images of the 10 wt % Cr-doped Al_2_O_3_ samples annealed at various temperatures from 1000 to 1600 °C for 6 h. As the annealing temperature increased, the Cr-doped Al_2_O_3_ exhibited colors ranging from green (samples a, b, c, and d) to gray (sample e) and red (samples g and h). The green color of the samples annealed up to 1200 °C was due to the presence of free Cr_2_O_3_. When the annealing temperature was 1400 °C or above, the Cr-doped Al_2_O_3_ tablet samples were obtained with red shades.

**Figure 2:**
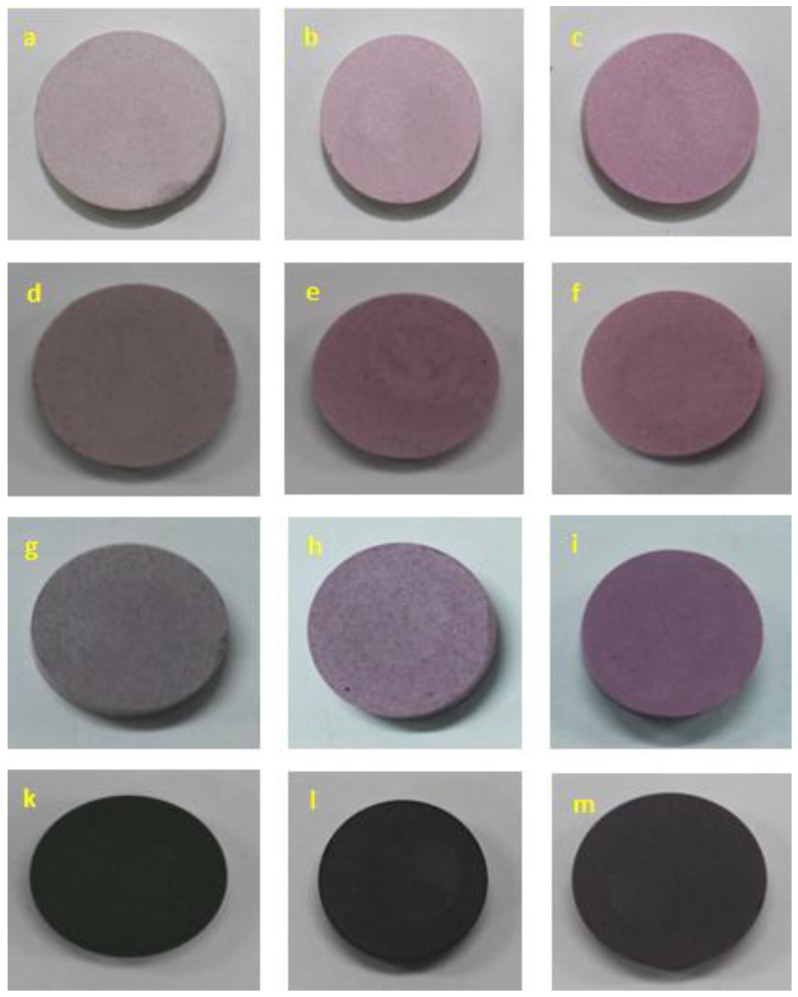
Images of Cr-doped Al_2_O_3_ doped with different chromium concentrations annealed at various temperatures : (**a**)–(**c**) 5 wt % Cr_2_O_3_ at 1400, 1500, and 1600 °C; (**d**)–(**f**) 10 wt % Cr_2_O_3_ at 1400, 1500, and 1600 °C; (**g**)–(**i**) 20 wt % Cr_2_O_3_ at 1400, 1500, and 1600 °C; and (**k**)–(**m**) 40 wt % Cr_2_O_3_ at 1400, 1500, and 1600 °C.

Figure 2 shows the images of the Cr-doped Al_2_O_3_ samples doped with chromium concentrations of 5, 10, 20, and 40 wt % annealed at temperatures from 1400 to 1600 °C for 6 h. As the annealing temperature increased from 1400 to 1600 °C, the color of the Cr-doped Al_2_O_3_ samples became brighter, i.e., from light pink (sample a) to pink (sample c), or from brown (sample d) to red (sample f). Moreover, the figure shows the effect of the chromium content on the color of the heated samples. As the chromium content increased, the color of the heated samples became darker, i.e., from pink (sample c) to red (sample f), dark red (sample i), and dark green (sample m), in this order. When the chromium concentration was between 5 and 20 wt %, the color of the synthesized samples was pink or red. However, the color of the samples doped with a chromium concentration of 40 wt % was dark green, regardless of the annealing temperatures (from 1400 to 1600 °C). This is because the ligand field changes with respect to the amount of chromium in the alumina. With the increase in the chromium concentration in the ruby, the color of the ruby changes from red to gray to green as the ligand field becomes weaker [37]. This result will be confirmed by the quantification of the color characteristics in the Section 2.4

### 2.2. Structural Characterization

#### 2.2.1. X-ray Diffraction (XRD) Spectra

**Figure 3:**
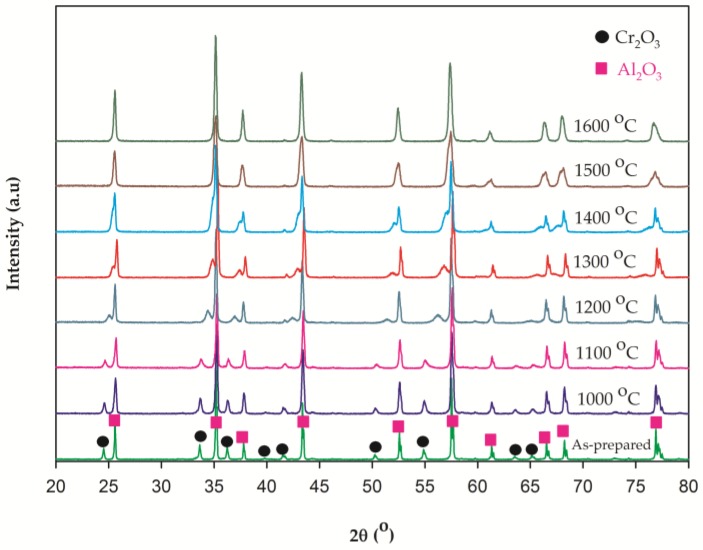
XRD patterns of 10 wt % Cr-doped Al_2_O_3_ samples before and after annealing at temperatures ranging from 1000 to 1600 °C for 6 h.

The compounds annealed at temperatures ranging from 1000 to 1600 °C were analyzed using XRD. Figure 3 shows the XRD patterns of the 10 wt % Cr-doped Al_2_O_3_ compounds annealed at 1000, 1100, 1200, 1300, 1400, 1500, and 1600 °C for 6 h. As the annealing temperature increased from 1000 to 1600 °C, the peak position of Cr_2_O_3_ shifted toward the peak position of Al_2_O_3_. The samples fired at 1000, 1100, and 1200 °C were green in color (Figure 1), and the XRD patterns exhibited separate diffraction peaks of the two crystalline phases (alumina-rich and chromia-rich solid solutions). This indicates that the doping reaction of Cr_2_O_3_ in alumina has not occurred. The doping reaction of Cr_2_O_3_ in the Al_2_O_3_ lattice commenced at an annealing temperature of 1300 °C. The XRD patterns of the samples fired at 1300 °C showed partially-overlapped diffraction peaks of both Cr_2_O_3_ and Al_2_O_3_. The color of the product changed to brown-green (Figure 1). A biphasic mixture was also observed after an annealing step performed at 1400 °C; however, the X-ray pattern showed a near unit phase. For annealing temperatures ranging from 1500 to 1600 °C, the disappearance of Cr_2_O_3_ diffraction peaks resulted in only the single phase being observed, implying Cr^3+^ uptake within the Al_2_O_3_ structure. This indicates that Cr^3+^ has been entirely incorporated into the Al_2_O_3_ lattice and uniformly substituted for Al^3+^ sites.

**Figure 4:**
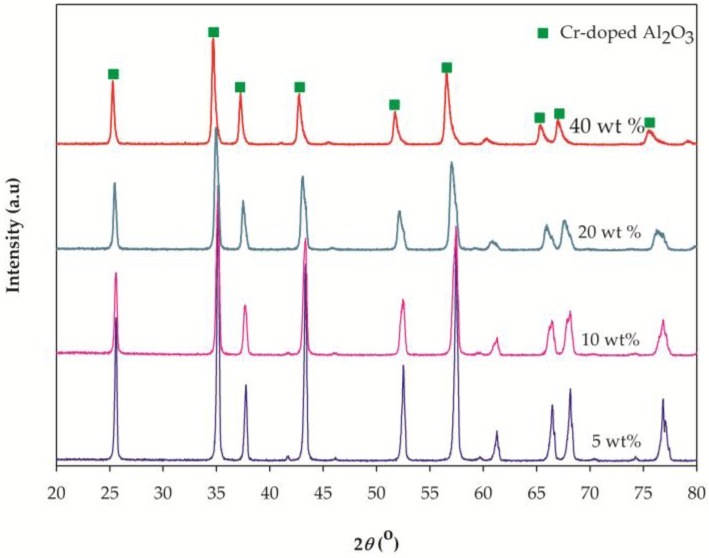
XRD patterns of Cr-doped Al_2_O_3_ doped with various chromium concentrations annealed at 1500 °C for 6 h.

Figure 4 shows the XRD patterns obtained for the Cr-doped Al_2_O_3_ compounds doped with different chromium concentrations at an annealing temperature of 1500 °C for 6 h. As the content of Cr_2_O_3_ increased, a slight shift in the XRD peak position toward lower 2*θ* values was observed (peaks position of Cr_2_O_3_). This phenomenon could be attributed to the larger size of Cr^3+^ ions with respect to Al^3+^ ions that led to expansions of the lattice [29]. In Figure 4, regardless of the chromium concentration, a single-phase Cr-doped Al_2_O_3_ was always obtained. This indicates that Cr^3+^ has been completely incorporated and substituted for Al^3+^ in the Al_2_O_3_ crystal lattice, regardless of the solid-solution composition. However, it could be observed that the increase in the chromium/aluminum ratio led to peaks broadening, so that a significant decrease in the relative intensity of the XRD peaks was observed. This could also indicate a decrease in the structural order.

#### 2.2.2. SEM Observations and Spot-Chemical Analysis.

**Figure 5:**
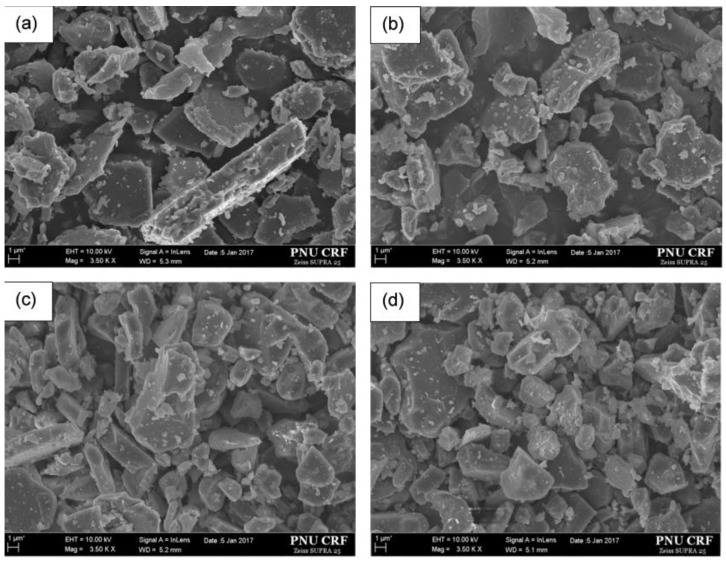
SEM micrographs of 10 wt % Cr-doped Al_2_O_3_ samples annealed at: (**a**) 1300 °C; (**b**) 1400 °C; (**c**) 1500 °C; and (**d**) 1600 °C for 6 h.

**Figure 6:**
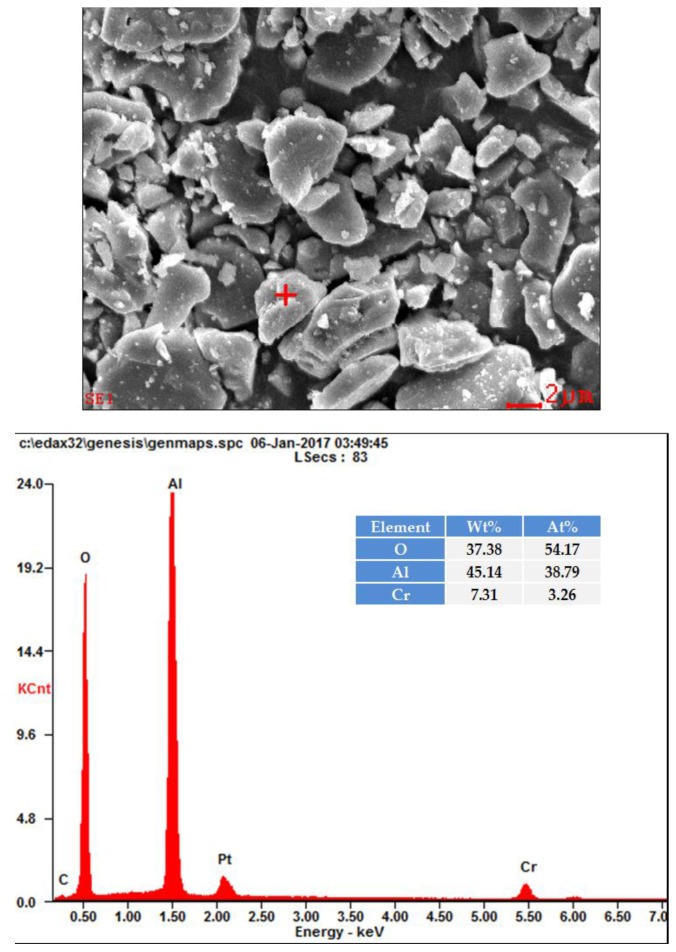
EDX result of 10 wt % Cr-doped Al_2_O_3_ sample annealed at 1600 °C for 6 h.

Figure 5 shows the SEM micrographs of the 10 wt % Cr-doped Al_2_O_3_ samples annealed at 1300, 1400, 1500, and 1600 °C for 6 h. The microstructures of the synthesized specimens showed that the particles were largely in cubic forms, and the particle sizes of the synthesized pigments were below 6 μm. The random spot-chemical analysis (EDX) conducted on the 10 wt % Cr-doped Al_2_O_3_ sample annealed at 1600 °C for 6 h (Figure 6) confirmed that the Cr^3+^ ions were present in the system. The table inserted in Figure 6 lists the element contents of the samples. The atomic and mass contents of the Cr^3+^ ions were 3.26% and 7.31%, respectively. The final mass content was close to the starting mass content.

#### 2.2.3. Fourier Transform Infrared Spectra (FT-IR)

**Figure 7:**
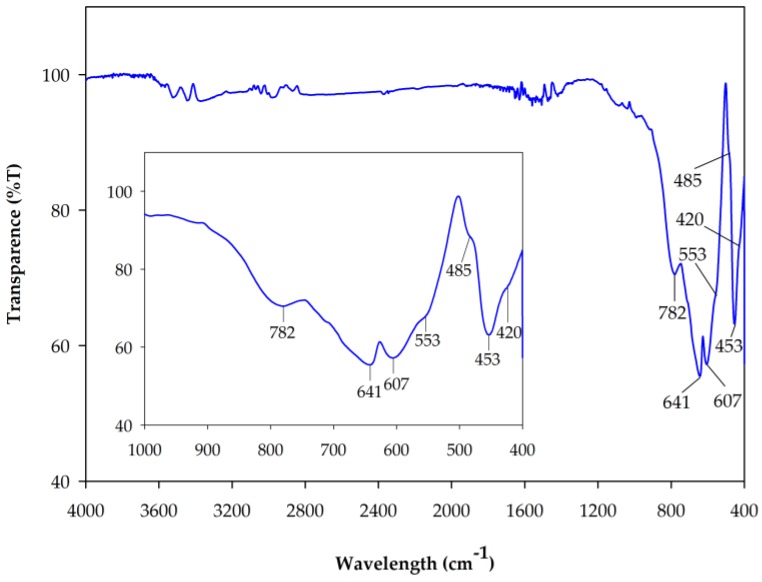
FT-IR spectrum of 10 wt % Cr-doped Al_2_O_3_ powdered sample annealed at 1600 °C for 6 h. The inset, corresponding to the spectrum of this sample, is shown on an enlarged scale.

In general, the FT-IR spectra of metal oxides show the peaks below 1000 cm^−1^ because of the inter-atomic vibrations [38]. Figure 7 shows a typical FT-IR spectrum of the 10 wt % Cr-doped Al_2_O_3_ calcined at 1600 °C for 6 h. The spectrum showed four strong peaks at 782, 641, 607, and 453 cm^−1^, and three weak peaks at 553, 485, and 420 cm^−1^. The observed peaks are in good agreement with the reported results [29,38,39]. It was reported that the bands at 786, 641, 595, 499, and 451 cm^−1^ were due to *α*-Al_2_O_3_ [26,40] and the bands at 638, 607, 547, 487, and 424 cm^−1^ were due to the stretching vibration of the Cr-O bonds [38]. Therefore, in this study, the peaks at 782, 641, 607, and 453 cm^−1^ are attributed to the Al-O stretching vibrations, and the peaks at 641, 607, 553, 485, and 420 cm^−1^ are attributed to the Cr-O stretching vibrations. This result indicates that the α-Al_2_O_3_ structure has formed with the Al-O stretching vibration of AlO_6_ octahedral structure, and confirms that the Cr_2_O_3_ is present in the system [26]. This finding is consistent with the XRD results.

### 2.3. UV-VIS Spectra

**Figure 8:**
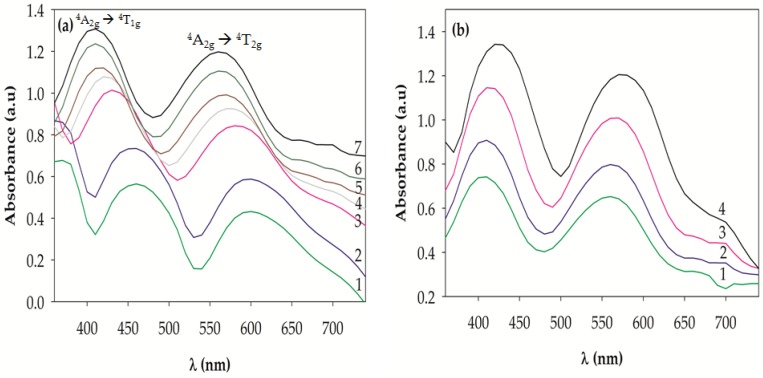
(**a**) UV-VIS spectra of the 10 wt % Cr-doped Al_2_O_3_ powdered samples annealed at (1) 1000 °C, (2) 1100 °C, (3) 1200 °C, (4) 1300 °C, (5) 1400 °C, (6) 1500 °C, and (7) 1600 °C for 6 h; (**b**) UV-VIS spectra of (1) 5 wt %, (2) 10 wt %, (3) 20 wt %, and (4) 40 wt % Cr-doped Al_2_O_3_ powdered samples annealed at 1600 °C for 6 h.

To study the effect of the annealing temperature on the vicinity of Cr^3+^ in the solid solutions, the 10 wt % Cr-doped Al_2_O_3_ powdered samples annealed at temperatures ranging from 1000 to 1600 °C were analyzed using the UV-VIS spectroscope. Figure 8a shows the absorption spectra. Two broad bands in the ranges of 410–461 nm and 561–600 nm were observed in the visible range. The two bands can be associated to the following d–d electronic transitions of Cr^3+^: ^4^A_2g_ → ^4^T_1g_ (410–461 nm), and ^4^A_2g_ → ^4^T_2g_ (561–600 nm) according to the diagram of Shirpour et al. [37]. The theory of the ligand field for a Cr^3+^ in an octahedral environment helps predict the existence of three absorption bands [41]. The energies of the first two electronic spins allowed the transitions ^4^A_2g_ → ^4^T_1g_(F) and ^4^A_2g_ → ^4^T_2g_(F) corresponding to visible light energies, whereas the third spin allowed the transition from ^4^A_2g_ to ^4^T_1g_(P) corresponding to ultraviolet light that does not affect the color. Depending on the ligand field created by the oxide ions, the position of these bands can be modified, resulting in synthesized samples with different colors. In fact, the two absorption bands of the pink Cr-doped alumina appear at 406 and 562 nm [29]. Figure 8a shows that as the annealing temperature increased from 1000 to 1600 °C, the two bands in the measured samples shifted to lower wavelengths (higher energy). This shift can be attributed to an increase of the ligand field with respect to Cr_2_O_3_ because of the decrease in the Cr–O distances resulting from the substitution of Al^3+^ (0.675 Å) by larger Cr^3+^ (0.775 Å) in the corundum structure [29,42]. Therefore, the color of the synthesized samples changed from green to pale gray, and to pink in appearance (Figure 1). In the absorption spectra of the samples annealed at 1500 and 1600 °C, the pink shade was associated with the absorption bands at 410 and 561 nm, which explained the existence of this coloration. In contrast, as the chromium concentration increased, the two bands of the measured samples shifted to higher wavelengths (lower energy), as shown in Figure 8b. This is because the ligand field changes with respect to the amount of chromium in the Al_2_O_3_ lattice. As the chromium content increases, the ligand field becomes weaker, thereby changing the color of the synthesized powdered samples from red to green (Figure 2) [37]. In the absorption spectrum, the two absorption bands of the 5 wt % Cr-doped Al_2_O_3_ samples appeared at 405 and 560.2 nm, which was in fairly good agreement with the reported results [29,43].

### 2.4. Color Property

**Figure 9:**
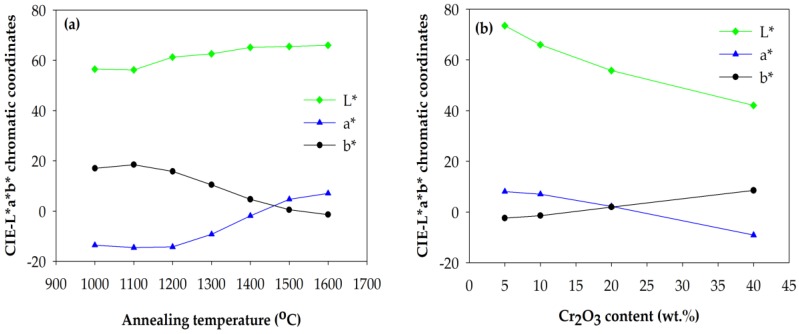
(**a**) Effect of annealing temperature on the CIE-L*a*b* color parameters of 10 wt % Cr-doped Al_2_O_3_ powdered samples annealed at various temperatures for 6 h; (**b**) effect of chromium content on the CIE-L*a*b* color parameters of Cr-doped Al_2_O_3_ powdered samples annealed at 1600 °C for 6 h.

Table 1 and Figure 9 present the CIE-L*a*b* coordinates of the Cr-doped Al_2_O_3_ powdered samples doped with different contents of chromium annealed at different temperatures. It could be observed that the highest red component (the highest value of coordinates a*) was obtained in the 5 wt % Cr-doped Al_2_O_3_ sample annealed at 1600 °C for 6 h. The brightness of the samples (L*) increased with the increase in the annealing temperature (Figure 9a) and decreased with the increase in the chromium content (Figure 9b). This is in fairly good agreement with the aforementioned results (Figure 2).

As the annealing temperature increased, the redness of the samples (a*) increased, while the greenness (b*) decreased (Figure 9a). In contrast, as the chromium concentration in the powdered samples increased, the greenness increased while the redness decreased (Figure 9b). This is because the ligand field changes with respect to the chromium content in the Al_2_O_3_ lattice. As mentioned previously, as the chromium concentration in the Cr-doped Al_2_O_3_ increases, the color of the synthesized samples changes from red to gray to green because the weakening of the ligand field results in the increase in the greenness and the decrease in the redness.

### 2.5. Thermochromism of Synthesized Pigment Powders

**Figure 10:**
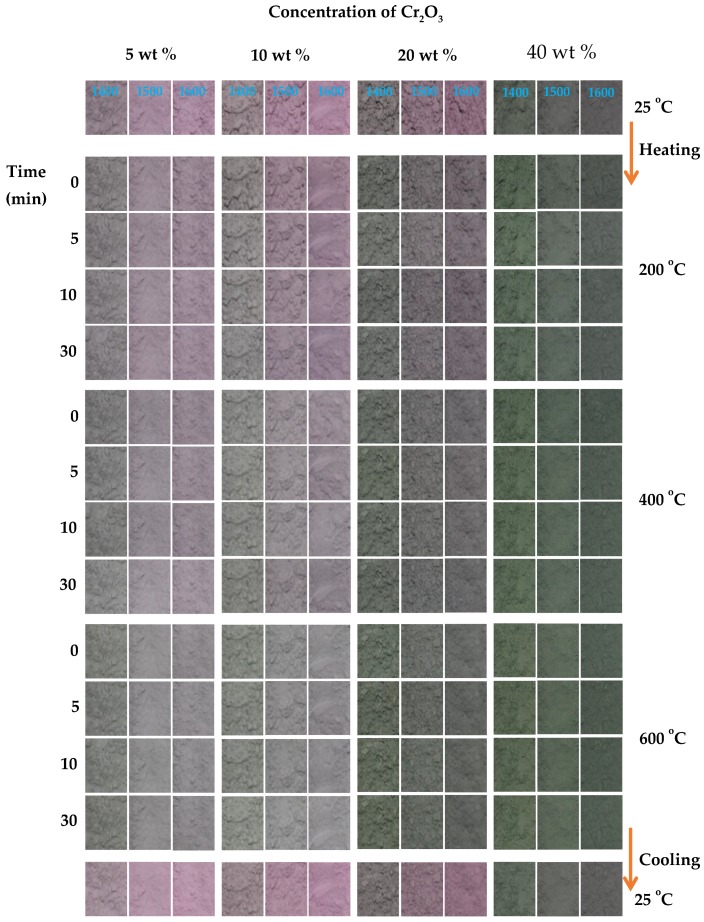
Color of Cr-doped Al_2_O_3_ powdered samples doped with various chromium contents annealed at 1400, 1500, and 1600 °C exposure at different temperatures for different times.

After synthesizing the Cr-doped Al_2_O_3_ and analyzing its structural properties, the thermochromism of the synthesized pigments was also studied, and the application of the pigments on the reversible thermochromic sensors was discussed. As described in the experimental section, the thermochromic color change of the synthesized samples at high temperature was investigated by adding the powdered samples into 5 mL combustion boats. Subsequently, the samples were heated to 200, 400, and 600 °C in an electric furnace, wherein the maximum temperatures were maintained for 0, 5, 10, and 30 min, respectively. After the Cr-doped Al_2_O_3_ powdered samples were exposed at a different temperature for different periods, the color was recorded at defined time points, as shown in Figure 10. The powdered samples underwent a color change from pink to gray/green when the temperature initially increased in the range of 25–600 °C. After cooling to room temperature, the color of the powdered samples returned to the original pink. This heating–cooling cycle can be repeated several times.

Figure 10 shows that the naked-eye-visible color transition of the Cr-doped Al_2_O_3_ depends on the temperature and chromium concentration in the Al_2_O_3_. At 200 °C, the change in color of the powdered pigments was insufficient to be observed by the naked eye, regardless of the chromium concentration. At higher temperatures, such as 400 and 600 °C, the color change from pink to gray/green could be observed most clearly in the samples annealed at temperatures above 1400 °C, as the chromium content ≤20 wt %. At higher chromium concentrations (40 wt %), the color change was insufficient to be distinguished by the naked eye because of the increase in the original green shade of the synthesized pigments and low optical contrast between the samples before and after heating. However, the color transition seemed to be independent of the exposure time at each maximum temperature. For instance, the color of the powdered samples after 30 min exposure at 600 °C was not significantly different from the ones after 0 min exposure. This indicated that the color change occurred immediately after the surrounding temperature reaches the maximum values, and further color modification was not observed, regardless of increasing the exposure time.

As mentioned previously, the color of the Cr-doped Al_2_O_3_ reversibly changed from pink to gray/green on heating at a pre-determined temperature depending on the Cr/Al ratio. The color change of the Cr-doped Al_2_O_3_ can be explained based on the ligand field theory of transition metal complexes [14]. The colors of these transition metal complexes are because of the d–d bands, or because the electronic transitions between the d-orbitals split under the electric field of the ligands. This splitting of the d-orbitals is the origin of the red color of the Cr-doped Al_2_O_3_. At low temperatures, a Cr^3+^ ion is being squeezed into an octahedral cage of O^2−^ ions in the Al_2_O_3_ lattice. At high temperatures, the chemical bonds in this compound expand, and the Cr^3+^ ions become more relaxed; thus, the green color is recovered, which is the color of pure Cr_2_O_3_ [14].

In summary, the results show that the thermochromic color change of the Cr-doped Al_2_O_3_ is reversible and is dependent on the temperature and concentration of chromium. Hence, the Cr-doped Al_2_O_3_ can be employed as a reversible thermochromic sensor at a temperature range of 25–600 °C.

## 3. Materials and Methods 

### 3.1. Synthesis

The thermochromic compound Cr-doped Al_2_O_3_ was prepared using the solid-state method. The raw materials were chromium oxide (Cr_2_O_3_ powder, ≥98% pure), aluminum oxide (Al_2_O_3_ powder, ≥98% pure), and acetone (99.5% extra pure, Samchun, Korea). The mixtures of the reactants were refined and homogenized in an agate mortar with acetone, and subsequently dried. After drying, the powders were pressed onto tablets using a disc-and-bar type mold under a uniaxial pressure of 25 MPa. First, to establish the temperature range for the thermal treatment of the samples, chromium oxide with a composition of 10 wt % (by weight) was chosen. The tablet samples were fired in an electric furnace at 1000, 1100, 1200, 1300, 1400, 1500, and 1600 °C with a heating rate of 5 °C/min and a soaking time of 6 h. The products were allowed to cool freely to room temperature in the furnace. The fired tablet samples were further hand-ground in an agate mortar using a pestle for 1 h to break the agglomerates and homogenize the particles. The red coloration characteristic of the Cr-doped Al_2_O_3_ pigment was obtained at T ≥ 1400 °C. Thus, in the next step, the effects of the Cr_2_O_3_ content on the structure, the degree of red coloration, and the color change at high temperatures of the Cr-doped Al_2_O_3_ pigments were investigated by adding various amounts of Cr_2_O_3_ (5, 20, and 40 wt %). The tablet samples were fired at 1400, 1500, and 1600 °C for a soaking time of 6 h at each maximum temperature.

### 3.2. Characterization Techniques

Phase analysis of the synthesized samples was performed using an X-ray powder diffraction (XRD) by employing a Rigaku MiniFlex600 diffractometer (Rigaku, Osaka, Japan) with Cu Kα radiation. The measurements were performed in a 2*θ* interval of 20°–80° with a step of 0.02° and a scanning rate of 2°/min. A goniometer was controlled using the MiniFlex Guidance’ software, which also helped determine the diffraction peak positions and intensities. The instrument was calibrated using an external Si standard.

The microstructures of the powdered samples were analyzed using a scanning electron microscope (SEM) (SUPRA^TM^ 25, Zeiss, Germany) coupled with an energy-dispersive X-ray spectrometer (EDX, AMETEK, New York, NY, USA). The following operating parameters were employed: measuring time of 100 s, working distance of 10 mm, acceleration of 15 KV, and count rates of 6200 cps. The samples for microstructural analysis were placed on an aluminum holder and were subsequently coated with platinum.

The Fourier transform infrared spectra (FT-IR) were recorded using a Nicolet IS5 FT-IR spectrometer (Thermo Fisher Scientific, Waltham, MA, USA). The powdered samples were mixed with dried KBr powder; subsequently, a force of approximately 25 MPa was applied to form the pellets. The spectra were collected over a spectral range of 4000–400 cm^−1^. 

The absorption spectra of the powdered samples were obtained using Konica-Minolta CM-3600d spectrophotometer (Konical Minolta, Japan) equipped with an integrating sphere coated with polytetrafluoroethylene (PTFE). The measurements were performed for wavelengths varying from 360 to 740 nm in the specular-component-excluded (SCE) mode. The CIE-L*a*b* chromatic parameters of the powdered samples were analyzed following the Commission Internationale de l'Eclairage (CIE) colorimetric method using the Konica-Minolta CM-3600d spectrophotometer (Konical Minolta, Japan) to quantify the color characteristics of the synthesized samples. A white calibration cap CM-A103 was used as the white reference and standard D_65_ (daylight) with a 10° observer angle (CIE1964) as the illuminant. In this method, L* indicates the brightness (L* = 0 for black and L* = 100 for white), and a* and b* are the chromatic coordinates (−a*, +a*, −b*, and +b* for green, red, blue, and yellow, respectively). Mathematical procedures were not required, as the chromatic parameters L^*^, a*, and b* could be obtained directly using the spectrophotometer.

The thermochromic color change of the powdered samples at high temperature was investigated by adding the powdered samples into 5 mL combustion boats, and subsequently, heating to 200, 400, and 600 °C in an electric furnace, wherein these maximum temperatures were maintained for 0, 5, 10, and 30 min, respectively. The color change of the powder samples was recorded using a digital camera (Canon EOS 100D, Tokyo, Japan)

## 4. Conclusions

An inorganic thermochromic material based on Cr-doped Al_2_O_3_ was successfully synthesized using the solid-state method and its thermochromic behavior was investigated. In this study, the Cr-doped Al_2_O_3_ exhibited a reversible color change from pink to gray/green as the temperature was varied in a range of 25–600 °C. This reversible color change depended on the temperature and concentration of chromium; however, it was independent of the exposure time. This novel property of the Cr-doped Al_2_O_3_ could be potentially applied to reversible thermochromic sensors at a temperature range of approximately 25–600 °C. These sensors could be used not only for warning users about damage due to overheating but also for monitoring the temperatures of various devices, such as aeronautical engine components, hotplates, and furnaces.

## Figures and Tables

**Table 1:** CIE-L*a*b* color parameters of Cr-doped Al_2_O_3_ powdered samples.

Cr_2_O_3_ Content (wt %)	Annealing Temperature T (°C)	L*	a*	b*
5%	1400	71.45	0.72	3.02
	1500	72.51	6.34	−1.05
	1600	73.48	8.1	−2.4
10%	1000	56.38	−13.56	16.97
	1100	56.12	−14.54	18.44
	1200	61.24	−14.26	15.78
	1300	62.57	−9.23	10.45
	1400	65.08	−1.89	4.65
	1500	65.43	4.68	0.47
	1600	65.94	7.03	−1.43
20%	1400	57.18	−6.24	7.43
	1500	55.85	−0.45	3.9
	1600	55.77	2.2	1.96
40%	1400	45.7	−13.04	11.86
	1500	43.7	−10.68	9.72
	1600	42.02	−9.09	8.48
